# Outcomes of direct anterior approach for uncemented total hip replacement in medial femoral neck fractures: a retrospective comparative study on the first 100 consecutive patients

**DOI:** 10.1186/s12891-023-06919-4

**Published:** 2023-10-02

**Authors:** Alberto Di Martino, Davide Pederiva, Matteo Brunello, Leonardo Tassinari, Giuseppe Geraci, Niccolò Stefanini, Cesare Faldini

**Affiliations:** 1 IRCCS - Istituto Ortopedico Rizzoli, Via Giulio Cesare Pupilli 1, Bologna, 40136 Italy; 2 Department of Biomedical and Neuromotor Sciences - DIBINEM, University of Bologna, Bologna, Italy

**Keywords:** Total hip arthroplasty, Proximal femur fractures, Direct anterior approach, Posterolateral approach

## Abstract

**Background:**

With the aging of the population, the incidence of medial femoral neck fractures is likely to increase, and along them the need for total hip replacement. The present study aimed to analyze whether the use of the direct anterior hip approach, compared with posterolateral approach in medial proximal femur fracture patients, results in any advantage in terms of complications rate and functional recovery.

**Methods:**

A total of 162 patients were included in the study, and divided by approach: 100 performed with direct anterior approach (group A) and 62 with posterolateral approach (group B). The two populations were overlapping in age (75 vs 74 years; *p* = 0.13), sex (58F 42M vs 46F 16M; *p* = 0.12) and BMI (24 vs 24; *p* = 0.77).

**Results:**

Group A showed a higher ASA score compared to group B (3 vs 2; *p* = 0.04). Similar hospital stays (7 vs 7 days; *p* = 0.55) and complication rates (6% vs 8%; *p* = 0.61) were observed among groups, the most frequent being periprosthetic fractures, and need for allogeneic blood transfusion (20% vs 13%; *p* = 0.25). Patients in group A (96 vs 85 min; *p* = 0.10) showed a slightly, longer surgical time and a faster postoperative functional recovery witnessed by the ability to climb stairs at hospital discharge (37% vs 21%; *p* = 0.041).

**Conclusion:**

The use of the direct anterior hip approach was effective in the management of frail patients with medial femoral neck fractures managed by total hip arthroplasty, allowing faster functional recovery in the elderly population.

## Background

Medial femoral neck fractures (MFNF) are among the most common and most feared fractures in the elderly [1], and their number is supposed to increase because of the aging of the population [2, 3]. Total hip arthroplasty (THA), when appropriate, is the recommended surgical treatment in patients able to walk independently and not cognitively impaired, given its more favorable long-term outcomes [4–7].

Several surgical approaches have been used for THA, direct lateral and posterolateral (PL) approaches being the most used in this setting [8–10]. Direct Anterior Approach (DAA) has been recently introduced into clinical practice to perform minimally invasive THA in patients with primary osteoarthritis, avascular necrosis of the hip and dysplasia, because of the advantages in terms of soft tissue sparing and faster postoperative recovery, with overall benefit for patient function [8]. DAA has already proven its efficacy in THA performance in the elderly, and it has been recently considered suitable to perform THA in fragile patients with MFNF [11].

At the Authors’ institution, DAA is the standard approach for THA, and recently it has been systematically introduced for the performance of THA for MFNF patients. Therefore, our study aims to research retrospectively whether there are benefits or potential complications of the use of the minimally invasive DAA to the hip for non-cemented THA, by the analysis of the clinical results of the first 100 consecutive MFNF patients. Data were compared to the historical cohort of patients operated by minimally invasive PL approach at the Authors’ Institution for the same fracture type.

## Methods

A retrospective study was conducted on patients operated on at the Authors’ Institution from May 1^st^, 2016 to March 31^st^, 2022 with an admission diagnose of proximal femoral fracture. The patients’ charts were screen independently by two surgeons to identify non-cemented THA following MFNF and then cross-checked by a third surgeon to minimize selection bias. The patients were divided according to the surgical approach used to perform the surgery and included minimally invasive DAA (according to Faldini et al. [12]) (group A) and minimally invasive PL approach [13]. While patients operated on through DAA were operated on since January 1^st^, 2019, all the PL approaches were performed before December 31^st^, 2019. All the procedures were performed by experienced senior surgeons proficient in THA both with a DAA and a PL approach.

Patients operated on for hemiarthroplasty of the hip, those operated on for elective or traumatic disorders other than MFNF, those with cemented implants, and patients operated on using other hip approaches (i.e. anterolateral or direct lateral) were excluded from the study. THA was performed in all patients with a life expectancy of more than 2 years who were able to move autonomously before the fracture.

For DAA, the patient was placed supine on a dedicated traction table and the incision was about 7cm long starting 2cm distal and lateral to the ASIS (anterior superior iliac spine) toward the fibular head; the interval between sartorius and tensor fascia lata was developed to the capsular plane. For PL approach the patient was in lateral decubitus on the operating table and the incision started 5cm proximal and 2cm posterior to the greater trochanter passing over it and continuing down the femoral shaft; after sharp dissection of the fascia and gluteus maximus muscle the short external rotators are reflected exposing the capsular plane. The day after the procedure each patient was assigned a physiotherapist with the objective of regaining functional independence through targeted exercises.

All implants were non-cemented; for the DAA these included Versafit or MPACT cups (Medacta, Suisse), and all the stems were AMISTEM or QUADRA (Medacta, Suisse). Cups used in PL approach were R3 (Smith&Nephews) or MPACT (Medacta), while stems were ADR (Smith&Nephews) or AMISTEM (Medacta).

Study parameters were retrieved from patients’ charts and recorded; these included: age, gender, BMI, comorbidities and personal medication history, ASA score, length of hospitalization, duration of surgery, intra (fracture) and postoperative complications (infection, dislocation, loosening, fracture, associated morbidity), either in hospital or after-hospital discharge, allogeneic blood transfusion requirement, and degree of ambulatory autonomy at discharge, including ability to climb stairs. The post-discharge follow-up was performed through clinical visits scheduled at 1, 3, 6, 12 months and yearly visits thereafter.

The primary outcome was the functional independence of the patient, evaluated by the ambulatory autonomy at discharge, assessed by a trained physiotherapist assigned to the patient after the procedure. The secondary outcomes were surgical complications, operative duration, in-hospital stay and allogenic blood transfusion requirement.

Statistical analysis was performed using the statistical program STATA (version 12, Stata Corp., College Station, Texas). Distribution of variables was reported using means and standard deviations (SD) for normally distributed data, and medians and interquartile ranges for non-normally distributed data. Data were tested for normality by the Kolmogorow-Smirnow test, and quantile-quantile plots of dependent variables were performed. Variables with non-normal distribution were presented with median and interquartile range. Statistical analysis was performed using the Kruskal Wallis test for continuous variables and the Fisher Exact test for categorical variables. Statistical significance was set at *p* < 0.05.

## Results

From May 1^st^, 2016 to March 31^st^, 2022, 162 consecutive uncemented THA implants for medial femoral neck fractures were performed at the Authors’ Institution. Of these, 100 were performed by DAA (Group A), and 62 by PL approach (Group B) (Table 1).

**Table 1:** Cohort characteristics and outcomes of Total Hip Arthroplasty through the DAA and PL approach in medial femoral neck fractures

	**DAA Approach**	**PL Approach**	***P*-value**
**Numerosity**	100 patients	62 patients	
**Sex**	58F - 42M	46F - 16M	0.12
**Age**	75 years (iqr 67–82)	74 years (iqr 69–78)	0.13
**BMI**	24 (iqr 22–24)	24 (iqr 22–24)	0.77
**ASA**	3 (iqr 2–3)	2 (iqr 2–3)	0.04
**Follow-up**	15 months (iqr 12–22)	60 months (iqr 48–72)	< 0.001
**Surgical time**	96 min (iqr 82–120)	85 min (iqr 64–92)	0.10
**In hospital stay**	7 days (iqr 6–9)	7 days (iqr 6–9)	0.55
**Allogeneic blood transfusion**	20%	13%	0.25
**Recovery of ambulation**	Walking: 93%	Walking: 94%	0.89
	Climbing stairs: 37%	Climbing stairs: 21%	0.041
**Complication rate**	6%	8%	0.61
**Surgery related**	3 femoral fractures (wiring)	1 femoral fracture (wiring)	0.19
	1 infection (superficial debridement)	1 infection (antibiotic therapy)	
**Postoperative**	1 infection (1-stage revision)	1 infection (2-stage revision)	0.58
	1 cardiovascular shock	2 cardivascular complications	

*Iqr* Interquartile range

Group A had an average follow-up of 15 months (range 4–24) and group B had an average follow-up of 60 months (range 30–77).

No significant differences were found in terms of age (*p* = 0.13), sex (*p* = 0.12) and BMI distribution (*p* = 0.77). Group A consisted of 58 female and 42 male patients averaging 75 years (IQR 67–82), with a BMI of 24 (IQR 22–26). Group B consisted of 46 females and 16 males with a median age of 74 years (IQR 69–78) and a median BMI of 24 (IQR 22–26).

Patients operated on by DAA showed a more severe ASA score at surgery, with a median value of 3, compared to a median of 2 for those patients operated by PL approach (*p* = 0.04). Surgery in DAA patients tended to be longer in DAA compared to PL patients, but no significant difference was observed (*p* = 0.098). In DAA patients surgical time averaged 96 min (IQR 82–120), and 85 min (IQR 64–92) for those operated by PL approach.

**Fig. 1:**
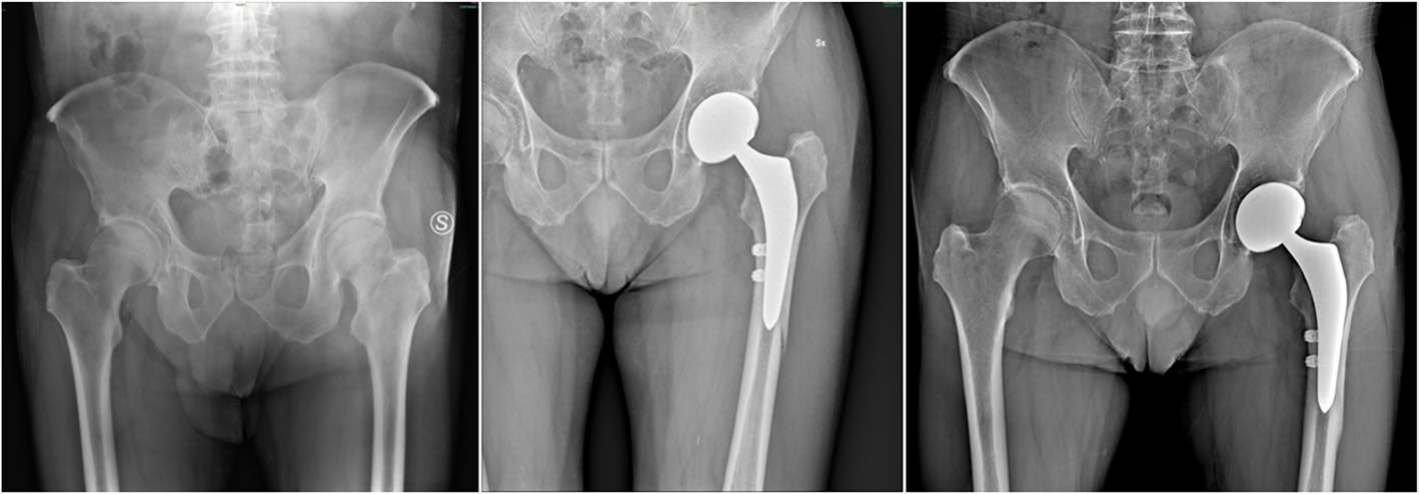
Pre and postoperative X-ray of a patient with femoral neck fracture undergoing total hip replacement through direct anterior approach. A periprosthetic femoral fracture was observed during the impaction of the stem and was managed by intraoperative wiring; at 12-month follow-up, complete fracture healing and bone remodeling were observed

As regards surgery-related complications, in group A, 4 perioperative complications were observed, including 3 intraoperative femur fractures and 1 infection. The three fractures were managed by intraoperative wiring (Fig. 1), while the infection, being superficial, was managed by surgical wound revision. In group B, 2 perioperative complications occurred: 1 femur fracture managed by wiring and 1 infection, and both did not require revision surgery. No dislocations were detected in the two groups. Statistical analysis showed no significant differences in the rate of complications between the two groups (*p* = 0.192).

Postoperative complications included 1 deep infection and 1 cardiovascular shock in the DAA population, and 1 deep infection and 2 cardiovascular complications in the PL group (*p* = 0.583). Both deep infections required implant revision.

There were no significant differences in the number of allogeneic blood transfusions performed for postoperative anemia, with 20/100 (20%) patients transfused in group A and 8/62 (12.9%) in group B (*p* = 0.246).

The average length of in-hospital stay showed no significant differences between the two groups, with a median of 7 days (IQR 6–9) for both groups (*p* = 0.552).

As regards the postoperative return to function while at hospital according to the physiotherapists’ charts, 2 patients in group A and 5 patients in group B had missing data. Of the remaining patients, no differences were found in terms of ability to ambulate. At discharge, 93% of patients in group A and 94% in group B ambulated with crutches or aids (*p* = 0.891). More patients operated on by DAA were able to climb stairs compared to PL patients (*p* = 0.041); in particular, 37% (36/98) of the patients in group A were able to climb stairs independently with aids or with the assistance of a physical therapist, compared to 21% (12/57) of patients in group B.

## Discussion

The current retrospective study investigated the outcomes of 162 patients operated on for uncemented THA for medial femur fractures. Of these, 100 were operated on by DAA approach (group A), and 62 by PL approach (group B). No differences in age, gender or BMI were detected comparing the two groups, while patients in group A showed a significantly higher ASA score at surgery, picturing a more fragile population composing group A. Patients in the two groups did not show any difference in terms of length of hospitalization, incidence of perioperative complications and ability to walk with aids at discharge. However, patients in the DAA group were more functional at discharge, as witnessed by their better ability to climb stairs.

The present study certainly has limitations. First, the limited follow-up of patients in group A and the limited sample size may underestimate the rate of reported complications. Second, the retrospective nature of the study, without validated clinical scoring, introduces with potential selection bias. Finally, the number of patients included in the study was not the work of a power analysis, thus underestimating any statistical significance.

The implementation of DAA in the performance of THA in fragile patients is not novel [14], and it has been effectively used to improve the outcomes of the procedure in elderly patients by decreasing surgical damage to soft tissues. However, the use of DAA to perform THA in the trauma setting for patients with MFNFs is relatively new, and only a few reports are available up to date [15–19].

Thürig et al. [15], reported the clinical and functional outcomes of 86 MFNF patients managed by THA through DAA. They reported that, despite DAA requiring a longer learning curve, the preservation of abductor muscles was associated with an advantage for elderly patients in terms of early recovery. Spina et al. [16], compared the outcomes of DAA with direct lateral approach for THA. The 69 patients operated by DAA showed less residual pain, faster recovery of ambulation and lower mortality, at the cost of a higher complication rate (10% v 2%, *p* = 0.046). Dimitriou et al. [19], analyzed the outcomes of THA through DAA in 150 trauma patients. The results were comparable to our findings, with a complication and re-operation rate of 9.3% and 3.3%, respectively. Only one study so far has compared DAA with PL approach for THA for MFNFs [20], with 36 patients in the DAA group and 31 in PL one. Chung et al. [20] found a non-significant difference in operating time, blood loss and complications rate (5.5% vs 3.2%) between the two groups. They found that DAA was more effective in the recovery of ambulation compared to PL approach.

Current knowledge shows that DAA in THA overlaps other approaches, including PL and direct lateral approach, in terms of length of stay, need for allogeneic blood transfusions, and incidence of peri or postoperative complications [8, 10, 21, 22]. Our study confirms these findings also in the trauma setting.

In our study, the overall complication rate was very low in both groups (6% in group A and 8% in group B). These numbers were significantly lower compared to the literature regarding THA surgery for MFNF [6], which reports an incidence of up to 16% of patients [23]. No dislocations were observed in our cohort, one of the most feared complications in post-traumatic THA [23–26] with rates up to 10% of patients [27]. No patients in our cohort had the implant of a dual mobility cup to reduce the risk of dislocation [25, 28]. The reduced sample size may have underestimated the risk of dislocation in both groups; however, the superiority of DAA in reducing the risk of post-operative dislocations compared to the PL approach has already been demonstrated [29–31].

The most common complications that were observed in our study population included intraoperative fractures and postoperative infections. We had a 3% fracture rate in group A and 1.6% in group B, both well below the values reported in the literature for elderly patients [2, 5]. All our implants were non-cemented, considering that at present cementation at the Authors’ institution is performed only for hemiarthroplasty implants; cementation itself is associated with cardiopulmonary risks, often with fatal consequences, especially in fragile patients [32, 33]. Intraoperative mortality after cementation reaches 0.1%, a little but significant number when related to the overall number of neck fractures occurring in elderly patients, a population with poor preexisting physical reserve and often sarcopenia [34]. Finally, the use of cement is associated with a longer operative time [35]. Data in the literature, despite emphasizing a higher prevalence of intraoperative fractures for uncemented prostheses, especially in osteoporotic patients [36], showed no prevalence of aseptic loosening in elderly patients undergoing uncemented total hip replacement [37]. In balancing the consequences associated with cementation and the risk of intraoperative fractures associated with the use of press-fit implants, it was decided at the Authors’ institution to promote the use of uncemented implants in the trauma setting as well.

The performance of DAA in our patients was associated with a non-significant, but slightly longer, surgical time [10]. Substantial overlap among the two groups occurred also for the number of allogeneic blood transfusions, with a non-significant slightly higher number of transfusions in group A, which can be correlated with the patients’ greater frailty and longer surgical duration [38]. Similarly, the overall revision rate during the follow-up was 1% in Group A and 1.6% in Group B. These values are lower compared to those available in the literature; in fact, recent evidence [5, 24] showed a 5-year cumulative incidence of revision of 4–8% in THA performed for femur fractures.

Functional recovery promotion is one of the main reasons to perform DAA in the elderly population [8, 39], and our study is in agreement with previous literature on the topic. Patients in group A, despite the greater overall frailty demonstrated by the higher preoperative ASA score, had an ambulatory recovery comparable to patients in group B, but DAA patients were more likely to climb stairs at discharge compared to PL patients. Although this finding could be considered of secondary importance in elective THA surgery, in patients affected by MFNF, a more rapid verticalization, ambulatory and functional autonomy recovery are related to an improvement in quality of life and reduced complications related to bed rest, with an increase in overall survival [40].

Seen that DAA for THA in MFNF is still a relatively recent surgical indication, it will be our endeavor to address these three limitations in the future. Finally, the Authors’ institution has a high volume DAA performance for THA implants, and these results could not be easily repeated in other institutions with less expertise in DAA performance.

## Conclusions

In conclusion, in surgical centers with a high volume of DAA for THA, the performance of the DAA in patients with MFNF is a valuable option. It is associated with an acceptable rate of complications and faster functional recovery compared to PL approach. Prospective multicentric studies are required to confirm current findings on a larger scale.

## Data Availability

Not applicable.

## Abbreviations

THA: Total Hip Arthroplasty
DAA: Direct Anterior Approach
PL: Posterolateral
MFNF: Medial femoral neck fractures
